# Breaking barriers in pulmonary health of patients with metabolic diseases: senolytics and beyond

**DOI:** 10.3389/fragi.2026.1812724

**Published:** 2026-07-17

**Authors:** Michaela Rennerova, Marie Zaloudikova, Martin Haluzik, Sona Stemberkova Hubackova

**Affiliations:** 1 Centre for Experimental Medicine, Institute for Clinical and Experimental Medicine, Prague, Czechia; 2 Faculty of Science, Charles University, Prague, Czechia; 3 Department of Physiology, Second Faculty of Medicine, Charles University, Prague, Czechia; 4 Diabetes Centre, Institute for Clinical and Experimental Medicine, Prague, Czechia; 5 Institute of Medical Biochemistry and Laboratory Diagnostics, First Faculty of Medicine, Charles University and General University Hospital, Prague, Czechia; 6 Institute of Biotechnology, Czech Academy of Sciences, Prague, Czechia

**Keywords:** cellular senescence, chronic pulmonary diseases, inflammaging, metabolic syndrome, senotherapy

## Abstract

Aging is the primary risk factor for most chronic diseases and is accompanied by the progressive accumulation of senescent cells within tissues. While cellular senescence initially serves as a protective mechanism that limits the proliferation of damaged cells, its persistent presence contributes to tissue dysfunction through the secretion of a broad spectrum of inflammatory and profibrotic mediators. The resulting chronic low-grade inflammation, oxidative stress, immune dysregulation, and impaired regenerative capacity are increasingly recognized as hallmarks of age-related pathology. Chronic pulmonary diseases, including chronic obstructive pulmonary disease and idiopathic pulmonary fibrosis, increase markedly with age and are increasingly regarded as manifestations of accelerated lung aging. Their development and progression are further exacerbated by obesity and type 2 diabetes mellitus, two highly prevalent metabolic disorders characterized by chronic metabolic stress, mitochondrial dysfunction, systemic inflammation, and enhanced accumulation of senescent cells. Emerging evidence suggests that cellular senescence represents a common biological denominator linking metabolic and pulmonary disease. Through persistent inflammatory and profibrotic signaling, senescent cells establish a self-perpetuating cycle of chronic inflammation, extracellular matrix remodeling, fibrosis, endothelial dysfunction, and impaired tissue repair, thereby driving progressive deterioration of both metabolic and pulmonary function. The recognition of cellular senescence as one of the important drivers of both chronic pulmonary and metabolic diseases has stimulated growing interest in therapeutic strategies aimed at reducing senescent-cell burden or attenuating its detrimental effects. Current approaches include both novel senotherapies specifically targeting cellular senescence, as well as established therapies used in metabolic diseases that have recently been shown to exert senescence-modulating effects. Although clinical evidence remains limited, targeting cellular senescence offers a unique opportunity to address the underlying biology of aging rather than individual disease manifestations. This review highlights the emerging role of cellular senescence as a mechanistic link between chronic pulmonary diseases and metabolic disorders and discusses its potential as a therapeutic target.

## 1 Introduction

Chronic pulmonary diseases (CPDs), including chronic obstructive pulmonary disease (COPD), idiopathic pulmonary fibrosis (IPF), asthma, and interstitial lung diseases, represent a significant global health burden. In recent decades, the prevalence of respiratory diseases has been steadily rising, driven by factors such as air pollution, unhealthy lifestyle and dietary habits, and exposure to harmful environmental agents. According to the World Health Organization (WHO), approximately 12 million people worldwide are diagnosed with CPDs annually (Ferkol and Schraufnagel, 2014).

CPDs are characterized by persistent respiratory symptoms and airflow limitation, which severely impact patient’s quality of life and lead to increased morbidity and mortality. Recent epidemiological studies have highlighted the complex interplay between CPDs and systemic conditions such as obesity and type 2 diabetes mellitus (T2DM). Both obesity and T2DM have reached epidemic proportions worldwide, contributing not only to a range of cardiovascular and metabolic disorders but also significantly exacerbating respiratory conditions. The impact of obesity and T2DM on the incidence, progression, and severity of CPDs has therefore become an important area of research. Studies indicate that individuals with obesity and T2DM often experience more severe forms of CPDs, leading to increased healthcare utilization, frequent hospitalizations, and greater economic burden. The coexistence of metabolic and pulmonary disorders presents a unique challenge, as patients often require integrated approaches to manage their respiratory and metabolic health effectively. Understanding the mechanisms through which these systemic conditions impact pulmonary health can inform clinical practices and public health strategies aimed at mitigating risks.

The relationship between CPDs and obesity/T2DM is bidirectional. Metabolic disorders can exacerbate respiratory symptoms, whereas CPDs may promote the development of obesity and T2DM through reduced physical activity, systemic inflammation, and progressive functional decline. This cycle of morbidity complicates treatment strategies and underscores the need for comprehensive, multidisciplinary approaches to care.

In light of the increasing prevalence of obesity and T2DM globally, particularly among vulnerable populations, there is an urgent need for further research to elucidate the mechanisms underlying the associations between these conditions and CPDs. Cellular senescence, as an integral part of the aging process, is one of the key factors influencing the development and progression of both metabolic diseases and CPDs. Senescent cells are characterized by cell cycle arrest and the production of various factors collectively termed the “senescence-associated secretory phenotype” (SASP), which may contribute to chronic inflammation, tissue damage, and the development of pathologies of metabolic and respiratory diseases (Barnes, 2017). Targeting cellular senescence therefore represents a potential multidisciplinary strategy in managing these interconnected conditions. This strategy becomes increasingly relevant due to the aging population, the rising prevalence of these diseases, and the need for new effective therapeutic approaches that focus on addressing the primary problem rather than merely suppressing pathophysiological phenomena. Recently, a new therapy aimed at eliminating senescent cells using senolytic agents has emerged, offering different levels of efficacy and use. These agents have the potential to influence the progression of diseases associated with aging and improve the quality of life of affected individuals (Wissler Gerdes et al., 2020).

## 2 Senescence

**FIGURE 1:**
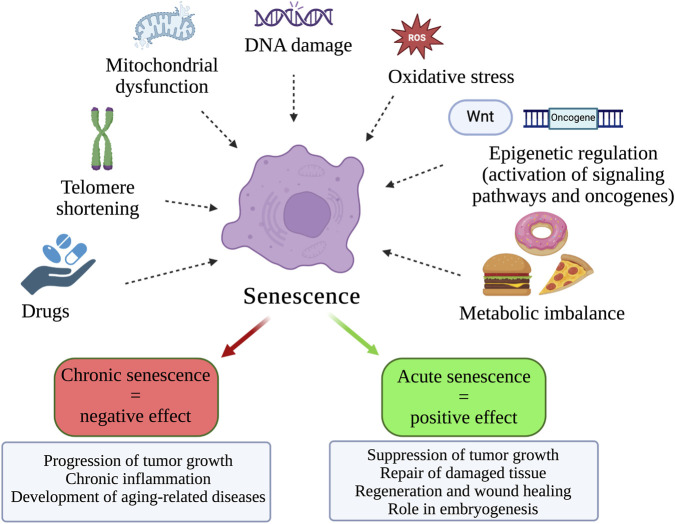
The emergence and consequences of senescence (created in BioRender.com).

Almost 60 years ago, L. Hayflick described a process that limits the proliferation of cells and termed it cellular senescence (Hayflick, 1965; Hayflick, 1968). In contrast to the latent proliferative potential of postmitotic quiescent cells, which play a key role in normal physiology and tissue homeostasis, cellular senescence represents a well-established anti-cancer defense mechanism forcing damaged and potentially oncogenic cells into cell cycle arrest. Senescence can be triggered in different cell types and tissues in response to diverse forms of cellular stress. Depending on the type of stress, we distinguish between naturally occurring senescence, found in cells with exhausted replicative potential (replicative senescence), and premature senescence, which occurs in cells exposed to external or internal stress (Figure 1). Replicative senescence is primarily triggered by the telomere shortening accompanying cell proliferation. Critical telomere shortening activates DNA damage responses leading to cell cycle arrest, which prevents uncontrolled growth and transformation into tumor cells (Hemann et al., 2001; Fumagalli et al., 2012). This type of senescence occurs during the natural aging of the organism. In contrast, premature senescence is induced by a variety of external and internal stressors. These include ionizing radiation, alterations in tissue pH or temperature, hypoxia, nutritional imbalance, oxidative stress, epigenetic alterations, or oncogene activation. It can also arise as a result of exposure of cells to chemicals and drugs, including therapies used in clinical practice. Many anticancer therapies induce DNA damage not only in malignant cells but also in surrounding healthy tissues, thereby triggering proliferation arrest and cellular senescence (Galbiati et al., 2017; Sachs et al., 1992; Quinlan et al., 2013; Serrano et al., 1997).

While short-term induction of cellular senescence can be beneficial in various settings, its long-term retention appears to be deleterious to the organism. Senescence is therefore divided into two main forms–acute (physiological) and chronic (pathological) (Figure 1). Acute senescence represents a well-timed and controlled response of the organism to specific stimuli, including the subsequent elimination of senescent cells. The immune system recognizes senescent cells through a combination of altered molecular signatures on the senescent cell and a robust multi-cellular response. Senescent cells are characterized by changes in their surface (upregulated stress ligands and altered MHC expression) and by their secretory phenotype (pro-inflammatory factors that recruit immune cells). Innate immune cells, especially natural killer (NK) cells, are the first responders that directly target senescent cells for lysis or phagocytosis, using receptors like Nature Killer Group 2D (NKG2D) and opsonin to detect these cells (Sagiv et al., 2016). Under physiological conditions, NK cells eliminate senescent cells *via* perforin- and granzyme-dependent cytotoxic mechanisms (Sagiv et al., 2013). However, chronic inflammatory conditions associated with aging and age-related diseases can progressively impair NK-cell function. Persistent exposure to inflammatory mediators and SASP components, especially TGF-β, promotes NK-cell exhaustion, alters their phenotype, and reduces expression of activating receptors such as NKG2D, thereby diminishing cytotoxic activity (Viel et al., 2016). Reduced NK-cell function impairs the efficient elimination of senescent cells, allowing their accumulation within tissues and further amplification of SASP-mediated inflammation [summarized in (Judge et al., 2020)]. This establishes a self-perpetuating feedback loop in which chronic inflammation promotes immune dysfunction, impaired senescent-cell clearance, and progressive tissue damage.

Additionally, adaptive immunity then provides support by secretion of cytokines (mainly CD4^+^ T cells) and direct elimination of senescent cells presenting antigens or expressing NKG2D ligands (mainly CD8^+^ T cells) to boost innate clearance mechanisms. All these mechanisms prevent the long-term accumulation of senescent cells, thereby suppressing cancer and limiting pathological fibrosis (Van Deursen, 2014). However, senescent cells are capable to remove immune-activating ligands from their surface and activate immune-inhibitory checkpoints, which makes them invisible to the immune system (Muñoz et al., 2019). Moreover, while acute SASP recruits immune cells to clear senescent cells, chronic SASP exposure has deleterious effects on immune cells. Persistently elevated IL-6 and IL-1β promote systemic inflammation (inflammaging), skew CD4^+^ T cells toward Treg and TH2 phenotypes, and inhibit TH1-driven cytotoxic responses. Prolonged SASP signaling also reduces CD8^+^ T-cell cytotoxicity, and drives immune cell exhaustion (so called immunoaging). Consequently, senescent cells accumulate within tissues, promoting chronic senescence. This process contributes to inflammaging, progressive tissue damage, and the development of age-related diseases (Wang et al., 2011).

Senescent cells exhibit resistance to apoptosis, partly due to the increased expression of BCL-2 family proteins. These proteins inhibit mitochondrial pathways leading to programmed cell death, allowing senescent cells to persist and accumulate in tissues (Yosef et al., 2016). Beyond cell-cycle arrest and SASP production, senescent cells acquire distinct phenotypic and subcellular characteristics that contribute to their persistence and pathological effects. Morphologically, senescent cells commonly exhibit altered cell architecture characterized by changes in cell size and cellular flattening accompanied by extensive cytoskeletal remodeling and altered intracellular organization. These changes are frequently associated with nuclear enlargement and altered nuclear morphology, often accompanied by increased cellular ploidy resulting from aberrant cytokinesis (Nishio and Inoue, 2005; Davoli et al., 2010). Alterations in nuclear structure also include loss of lamin B, a structural component of the nuclear envelope, which contributes to chromatin reorganization and facilitates expression of SASP-associated genes (En et al., 2020).

Senescent cells display marked metabolic alterations characterized by mitochondrial dysfunction, increased mitochondrial mass, and elevated production of reactive oxygen species (ROS), which promote DNA damage and reinforce senescence-associated signaling pathways (Wiley et al., 2016). Persistent DNA damage responses, driven not only by mitochondrial dysfunction, frequently lead to the formation of irreparable DNA damage foci and altered chromatin architecture, including senescence-associated heterochromatin foci (SAHF), which contribute to stable silencing of proliferation-related genes (Kosar et al., 2011). Furthermore, increased expression and accumulation of promyelocytic leukemia protein (PML) nuclear bodies have been detected in senescent cells and are thought to participate in DNA damage signaling and maintenance of the senescent phenotype (Pearson et al., 2000; Ma et al., 2023).

Another characteristic feature of senescence is expansion of the lysosomal compartment and increased lysosomal activity, commonly reflected by elevated senescence-associated β-galactosidase (SA-β-gal) activity, one of the most widely used markers of senescence (Lee et al., 2006). Increased lysosomal content likely reflects enhanced processing of damaged organelles, unfolded proteins, and cytoplasmic chromatin fragments generated during senescence development. However, prolonged lysosomal stress may ultimately impair degradative capacity and disrupt cellular proteostasis, promoting accumulation of damaged macromolecules and dysfunctional organelles (Tai et al., 2017). Collectively, these alterations establish a self-reinforcing intracellular environment that maintains the senescent phenotype, amplifies SASP signaling, and contributes to tissue dysfunction and age-related pathologies.

SASP plays an important role in autocrine/paracrine signaling and maintenance of the senescent phenotype. Since SASP represents a response to cellular damage, one of its beneficial functions involves communication with immune cells through the production of pro-inflammatory cytokines that signal the damaged cell. However, prolonged SASP production profoundly affects the behavior of cells in the environment and contributes to the development of systemic inflammation and age-related diseases (Davalos et al., 2010; Soriani et al., 2009). SASP factors influence both neighboring cells through paracrine signaling and distant tissues through endocrine signaling. Paracrine/endocrine SASP signaling can activate NF-κB and C/EBPβ in healthy cells, leading to subsequent increased expression of inflammatory molecules and activation of the p53/p21^cip1/waf1^ and p16^INK4a^/pRb pathways, cell cycle arrest and the development of senescence. Thus, not only direct damage, but also long-term exposure to stress molecules activates responses that induce growth arrest and so-called secondary “bystander” senescence. This clarifies the mechanism by which senescent cells are able to increase their number and accumulate even in tissues not affected by the primary stress (Acosta et al., 2008; Acosta et al., 2013).

## 3 Respiratory diseases

Respiratory diseases comprise a heterogeneous group of disorders affecting the lungs, airways, and respiratory muscles. They can be caused by a variety of factors, such as infections (viral, bacterial, or fungal), environmental pollutants, smoking, genetic predisposition, autoimmune disorders, or occupational exposures. An etiological classification distinguishes infectious (e.g., bacterial pneumonia, tuberculosis) from non-infectious diseases (e.g., asthma, idiopathic pulmonary fibrosis). A temporal classification separates acute conditions (sudden onset, e.g., acute bronchitis or pneumonia) from chronic conditions (long-standing, e.g., COPD or chronic asthma). Additionally, an anatomical classification groups diseases by the primary site of pathology as diseases primarily affecting the airways (such as bronchial asthma or chronic bronchitis), the pulmonary vasculature (such as various forms of pulmonary hypertension), or the lung parenchyma and interstitium (such as emphysema, interstitial pulmonary fibrosis, or lung cancer) (Gould et al., 2023).

In clinical practice, these classification approaches are complementary and often overlapping. In many respiratory disorders, the pathological process is not confined to a single part of the system. Initial damage in one region can lead to secondary changes elsewhere. COPD is a classic example of a disease primarily affecting the airways and alveoli, with chronic bronchitis involving the bronchi and emphysema affecting the alveolar compartment. In advanced stages, COPD frequently leads to pathological changes in the pulmonary circulation. Chronic alveolar hypoxia in late-stage COPD triggers pulmonary vasoconstriction and vascular remodeling, which can culminate in secondary pulmonary hypertension. This secondary vascular complication exemplifies how a disease initially damaging the airways and air-exchange regions can subsequently injure the pulmonary vessels as well (Goel et al., 2022). Another example of overlapping pathology is the interplay between parenchymal lung damage and airway disease in conditions like bronchiectasis. Chronic infection and inflammation in the airways can distort lung architecture and lead to regions of fibrosis, while the altered lung mechanics further predispose to infection (King, 2009). Collectively, these examples illustrate that respiratory diseases often involve interconnected pathological processes extending beyond the primary site of injury.

Beyond direct injury from infections or toxins, aberrant repair mechanisms can themselves cause significant lung damage. In a healthy response to injury, inflammation subsides and normal tissue architecture is restored. However, if the reparative process is dysregulated or excessive, the outcome may be pathological fibrosis, a form of scarring that stiffens and disrupts lung function. Development of fibrosis after infection or inflammation illustrates how a physiological repair response can become maladaptive and ultimately contribute to chronic respiratory dysfunction. Given the diversity and overlapping nature of respiratory pathologies, the most pragmatic way to understand and classify a case is to identify its primary cause and the pathophysiological mechanism leading to the patient’s lung dysfunction.

**FIGURE 2:**
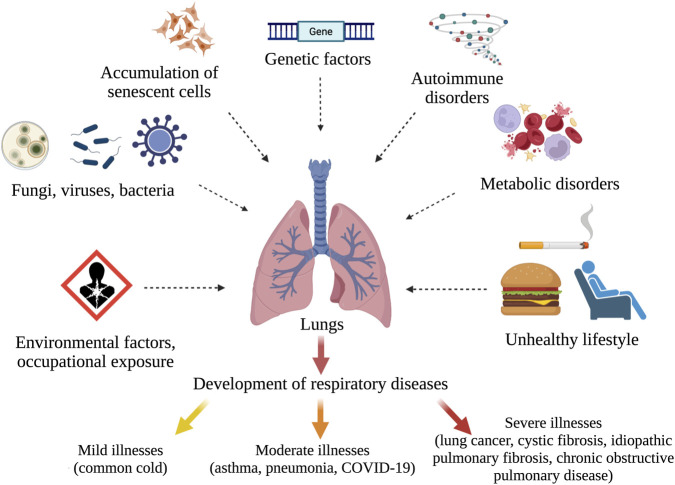
Factors contributing to development of respiratory diseases (created in BioRender.com).

Respiratory diseases can also be classified according to their clinical severity into: (i) mild conditions, such as the common cold; (ii) moderately severe diseases, including asthma and pneumonia or COVID-19; and (iii) severe disorders, such as lung cancer, cystic fibrosis, IPF, and COPD (Figure 2). While the first two groups are so-called acute respiratory diseases, the third group is classified as CPDs, which represent a significant health and financial burden on society worldwide. In Europe, the prevalence of CPDs ranges between 4%–10% of the adult population (Ferkol and Schraufnagel, 2014; Hutchinson et al., 2015).

Although the effects on a patient’s life are often devastating, there is still no effective treatment that can cure these diseases. Current therapies primarily alleviate symptoms and slow disease progression but rarely stop or reverse the underlying pathological process. Despite intensive research, the pathogenesis of CPDs is still not fully understood. Prevention, early diagnosis and a multidisciplinary approach are therefore important to ensure optimal treatment (Ferkol and Schraufnagel, 2014; Hutchinson et al., 2015).

Cellular senescence has emerged as an important contributor to the pathogenesis of IPF, COPD, and several other respiratory diseases (Torres et al., 2013). There is a positive correlation not only between the age of the patient and the incidence of these diseases, but also their severity. Aging is associated with structural and functional alterations of the respiratory system, including reduced lung elasticity, diminished chest wall compliance, and weakening of respiratory muscles. These changes may contribute to reduced lung function and respiratory capacity, and increased susceptibility to respiratory disease. With increasing age, the immune system also undergoes immunosenescence, which can lead to reduced immune cell function and an impaired ability to fight respiratory infections. The lungs of elderly individuals are also much more susceptible to harmful external influences, such as cigarette smoking or living in a highly polluted environment (Barnes, 2017).

### 3.1 Idiopathic pulmonary fibrosis

IPF is the most common type of idiopathic interstitial pneumonia occurring predominantly in elderly patients (60–70 years), and its incidence has been increasing in recent years (Hutchinson et al., 2015). It is a progressive, chronic, and irreversible disease characterized by extensive accumulation of fibrotic tissue in the lungs, leading to a gradual decline in respiratory function and in most cases resulting in death within 5 years of diagnosis (Meltzer and Noble, 2008). The pathogenesis of IPF reflects an interplay between genetic susceptibility and environmental exposures, including smoking, airborne dust, and industrial or chemical pollutants. The primary cause of IPF is thought to be recurrent micro-injuries to the lung epithelium. These epithelial lesions lead to fibroblast activation, triggering a wound healing response. In IPF, however, this repair process becomes dysregulated, resulting in excessive extracellular matrix (ECM) deposition and progressive fibrotic tissue formation (Ushakumary et al., 2021).

Although it is not primarily an inflammatory disease, in the past, patients were given anti-inflammatory therapies such as corticosteroids or immunosuppressants and cytostatics. However, these treatments did not improve survival, which is usually between 2 and 5 years post-diagnosis (Raghu et al., 2011). At best, the rate of decline in respiratory function was positively affected, but there was no significant improvement over placebo (Fernando and Martinez, 2014). Currently, the most widely used antifibrotic drugs are nintedanib and pirfenidone, which reduce fibroblast formation and proliferation, collagen production, and inhibit the inflammatory environment in the lungs. Both drugs have common side effects, but are usually well tolerated by patients (Loveman et al., 2015). In patients with advanced IPF, supplemental oxygen therapy is often prescribed to alleviate low blood oxygen levels. However, all current treatments only slow down the progression of lung failure but do not reduce mortality in patients with IPF (Hagmeyer et al., 2016).

Current studies show that senescence plays a key role in the pathogenesis of IPF. It is manifested by premature senescence of alveolar epithelial cells (AECs) due to shortened telomeres, which leads to their disruption, thereby limiting the regenerative mechanisms of lung tissue (Wasnick et al., 2023). The consequence is progressive remodeling of the lung parenchyma, driven by aberrant activation of the Wnt/β-catenin signaling pathway, structural deformation, proliferation of lesions that adversely affect the bronchi and fibrosis in the lung (Selman et al., 2008; Bridges et al., 2025). Activation of the Wnt/β-catenin pathway in senescent cells also leads to abnormal epithelial regeneration at bronchiolar-alveolar junctions, which can lead to a loss of alveolar epithelium on one side while causing an increase in abnormal bronchiolar epithelium on the other side (Chilosi et al., 2003; Königshoff et al., 2008; Aros et al., 2021). Inhibition of the Wnt/β-catenin pathway can reverse the fibrosis and therefore represents a promising therapeutic target in reducing the pathogenesis of IPF (Wasnick et al., 2023; Selman et al., 2008).

Production of SASP and free radicals by the senescent cells promotes a fibrotic environment and disrupts tissue homeostasis in the lungs (Coppé et al., 2010). This leads to inadequate immune regulation, an increased chronic inflammatory response and accumulation of immune cell in the lungs, further increasing overall inflammation (Marchal-Sommé et al., 2006; Valenzi et al., 2019). Especially alveolar macrophages (AM), playing a central role in maintaining pulmonary homeostasis, adopt maladaptive phenotypes that promote tissue injury. Chronic exposure to SASP shift AM from a reparative to a pro-inflammatory state, which results in the release of ROS, proteases, and additional cytokines that reinforce epithelial senescence and matrix degradation. This bidirectional crosstalk contributes to the pathogenesis of IPF (Cui et al., 2025). Emerging evidence also suggests that metabolic reprogramming within senescent AM, particularly altered mitochondrial function and lipid metabolism, further drives their pathogenic activity (Ryan et al., 2023).

Increased inflammatory response subsequently leads to the differentiation of fibroblasts into myofibroblasts, which hinder proper tissue regeneration by increasing the production of extracellular matrix that fills the lungs and replaces functional tissue. These myofibroblasts are therefore key effector cells in the pathogenesis of pulmonary fibrosis (Coppé et al., 2010). The accumulation of fibrotic tissue due to the increased presence of senescent cells may also explain the increased risk of lung cancer in patients with IPF (Koo et al., 2016; Takeuchi et al., 2010; Lei et al., 2025).

In addition to increased levels of pro-inflammatory factors, senescent cells also produce increased levels of factors such as vascular endothelial growth factor (VEGF), fibroblast growth factor (FGF), and matrix metalloproteinases (MMPs), which can promote angiogenesis and vascular remodeling by stimulating endothelial cell proliferation, migration, and formation. Disruption of the pulmonary vasculature due to abnormal angiogenesis and impaired endothelial function may contribute to the pathogenesis of IPF by altering oxygen delivery, promoting hypoxia, and stimulating fibroblast activation and ECM deposition (Ushakumary et al., 2021).

### 3.2 Chronic obstructive pulmonary disease

Like IPF, COPD affects up to 10% of the adult population (Ag et al., 2020). This disease causes breathing complications due to restricted airflow, which eventually develops into a chronic inflammatory response in the lungs. Compared with IPF, which predominantly affects older individuals, COPD is most commonly diagnosed after the age of 40, and its prevalence rises markedly with age. However, early-onset disease may occur in individuals with significant environmental exposures or genetic susceptibility (Adeloye et al., 2022). Six principal clinical phenotypes of COPD have been described: chronic bronchitis, pulmonary emphysema, exacerbation-predominant type, bronchiectasis-associated COPD, asthma–COPD overlap, and pulmonary cachexia. Among these, chronic bronchitis and pulmonary emphysema are the most prevalent.

In general, COPD is defined by airflow limitation and impaired dynamic spirometric parameters, yet the underlying mechanisms of these changes differ among phenotypes. In chronic bronchitis, the hallmark features include thickening of the bronchial wall and hypersecretion of mucus, resulting in airway narrowing and increased airflow resistance. Clinically, chronic bronchitis is diagnosed when a patient presents with productive cough lasting for at least 3 months in two consecutive years, provided other pulmonary or cardiovascular causes have been excluded (Kakavas et al., 2021).

In contrast, pulmonary emphysema is characterized primarily by destruction of alveolar walls, particularly the interalveolar septa, leading to the permanent enlargement of distal airspaces. The consequent loss of elastic recoil impairs passive expiration, so that exhalation must be more forceful, similar to the expiratory effort observed in chronic bronchitis with airway obstruction. Because both phenotypes share the common feature of chronic airflow limitation, they are grouped under the collective diagnosis of COPD (Tuder et al., 2006).

In advanced stages of the disease, chronic hypoxemia frequently develops and contributes to additional complications, most notably hypoxic pulmonary hypertension. This condition is driven by pulmonary vascular remodeling and a progressive increase in pulmonary vascular resistance, further exacerbating the cardiorespiratory burden associated with late-stage COPD (Bogaard, 2017).

In addition to respiratory symptoms, COPD is associated with numerous systemic complications (Fabbri and Rabe, 2007). One of the most common is weight loss resulting from skeletal muscle wasting, which further impairs physical performance and reduces quality of life. Additional comorbidities frequently associated with COPD comprise cardiovascular diseases, an increased risk of T2DM, osteoporosis, anemia, autoimmune disorders and depression, affecting the majority of patients with advanced or severe COPD (Agusti and Soriano, 2008).

Treatment of COPD usually involves a combination of pharmacological and non-pharmacological approaches aimed at relieving symptoms, improving lung function and improving quality of life. Bronchodilators, drugs that provide airway relaxation and dilation, are used as maintenance therapy to ensure airway patency and long-term improvement in symptoms and lung function. In patients with more severe COPD, they are used in combination with inhaled corticosteroids to help reduce airway inflammation. However, their use is associated with an increased risk of pneumonia and other side effects. An alternative to corticosteroids are phosphodiesterase-4 inhibitors, which also reduce airway inflammation (Tashkin et al., 2008). However, despite the undeniable positive effects for the patient, it is only a reduction of pathophysiological symptoms rather than a treatment of the cause of the disease.

Similar to IPF, cellular senescence is one of the key factors regulating lung tissue dysfunction in COPD patients (Coppé et al., 2010; Bogaard, 2017; Guo-Parke et al., 2025). Multiple cell populations within the lung undergo senescence in response to chronic oxidative stress and sustained inflammation. Among these, AECs are particularly vulnerable. Senescent AECs show impaired regenerative capacity and adopt a SASP, releasing proinflammatory cytokines and matrix-degrading enzymes that exacerbate tissue destruction (Parimon et al., 2023). Senescence of pulmonary endothelial cells leads to endothelial dysfunction, reduced nitric oxide bioavailability, and increased vascular permeability. These alterations contribute to pulmonary vascular remodeling and, in advanced disease, the development of hypoxic pulmonary hypertension (Van Der Feen et al., 2020; Noureddine et al., 2011). Fibroblasts and myofibroblasts in COPD lungs exhibit features of replicative senescence. Their reduced proliferative capacity and altered extracellular matrix production impair tissue repair and contribute to emphysematous remodeling (Yanai et al., 2015; Karakioulaki et al., 2020). Similarly, airway smooth muscle cells acquire a senescent phenotype, marked by abnormal proliferation and secretion of growth factors that perpetuate airway wall thickening and remodeling (Wrench et al., 2024). In parallel, senescent AMs maintain chronic low-grade inflammation through persistent release of ROS and cytokines, thereby amplifying the SASP network across the lung microenvironment (Prieto et al., 2023). Collectively, senescence of these diverse cell types sustains a self-perpetuating cycle of inflammation, impaired tissue repair, and progressive functional decline that underpins the pathogenesis of COPD.

The Wnt/β-catenin signaling pathway plays a role in the pathogenesis of both IPF and COPD. In contrast to IPF, Wnt/β-catenin signaling is suppressed in senescent lung parenchymal cells during COPD progression, which promotes the expression of MMPs. Inactivation of the Wnt/β-catenin pathway is a reversible mechanism that can be stimulated by TGF-β1, which may help alleviate COPD emphysema and promote lung tissue regeneration (Kneidinger et al., 2011). These observations highlight the context-dependent role of Wnt/β-catenin signaling in chronic pulmonary diseases. In IPF, persistent activation of Wnt/β-catenin signaling in alveolar epithelial cells and fibroblasts promotes aberrant epithelial repair, myofibroblast differentiation, extracellular matrix production, and resistance to apoptosis, thereby driving progressive fibrotic remodeling. In contrast, COPD and emphysema are characterized by reduced Wnt/β-catenin activity, particularly within alveolar epithelial progenitor cells, resulting in impaired epithelial regeneration and defective alveolar maintenance (Skronska-Wasek et al., 2017). Loss of Wnt signaling also favors matrix degradation through increased expression of matrix metalloproteinases and contributes to destruction of alveolar structures (Baarsma et al., 2017). Thus, while excessive Wnt/β-catenin activity promotes pathological tissue repair and fibrosis in IPF, insufficient pathway activation in COPD impairs regenerative responses and accelerates emphysematous tissue loss. These findings illustrate how dysregulation of the same signaling pathway can lead to distinct pathological outcomes depending on the cellular context and disease phenotype, emphasizing the need for disease-specific therapeutic approaches.

Sirtuin 1 (SIRT1) is a nicotinamide adenine dinucleotide (NAD+) dependent deacetylase involved in the regulation of cellular senescence [summarized in (Sun et al., 2023)]. SIRT1 plays an important role in the regulation of inflammatory and antioxidative processes. The reduction of SIRT1 levels in the lung parenchyma leads to increased production of pro-inflammatory cytokines due to activation of the NF-κB pathway (Rajendrasozhan et al., 2008). Additionally, reduced SIRT1 activity has been associated with activation of the p53/p21^cip1/waf1^ or p16^INK4A^/Rb senescence pathways, whereas SIRT1 activation exerts anti-senescent effects by promoting cellular stress resistance, maintaining mitochondrial function, and enhancing FOXO-mediated protective signaling (Rajendrasozhan et al., 2008; Houben et al., 2009).

SIRT1 also contributes to tissue repair and regeneration by regulating stem-cell maintenance, proliferation, and differentiation. Targeted activation of SIRT1 may enhance the regenerative capacity of lung cells, facilitating tissue repair in lungs damaged by COPD [summarized in (Li et al., 2023)]. In this regard, the use of SRT2104, a specific activator of SIRT1, appeared to be a promising treatment for COPD in preclinical studies, as this agent reduced emphysema formation in the lungs. However, its clinical development has encountered significant challenges, and robust data confirming its efficacy and safety in humans are still lacking (Yao et al., 2014; Rahman et al., 2012).

Despite intensive research, current treatment of CPDs, especially IPF and COPD, relies on pharmacological treatment and supportive care that focuses solely on slowing disease progression, alleviating symptoms and improving quality of life, but not at eliminating the primary cause of the disease. Therefore, future therapeutic strategies should focus on targeting the mechanisms that drive inflammation, oxidative stress, fibrosis, and cellular senescence. Given the central role of senescent cells in the pathogenesis of IPF and COPD, their selective targeting represents a promising strategy to slow disease progression and improve patient outcomes.

## 4 Relationship between metabolic syndrome and respiratory diseases

Metabolic diseases, including obesity, T2DM or dyslipidemia, have emerged as significant global health concerns over the past few decades. The increasing incidence and severity of metabolic diseases worldwide are alarming, with implications for healthcare systems, economies, and the overall wellbeing of populations. The WHO reports that the prevalence of obesity has tripled since 1975, with over 1.9 billion adults classified in 2020 as overweight (Body Mass Index–BMI >25) and more than 650 million as obese (BMI >30). This surge in obesity is closely linked to the rising incidence of T2DM, which affects over 400 million people globally. The relationship between obesity and T2DM underscores the multifaceted nature of metabolic diseases, as they often coexist and exacerbate one another. Both conditions are driven by lifestyle factors such as poor diet, physical inactivity, hectic lifestyle and irregular or insufficient sleep patterns, highlighting the need for comprehensive public health interventions (WHO Technical Report Series OBESITY, 2000).

The severity of metabolic diseases is not only a consequence of their prevalence but also of their associated comorbidities. Conditions such as cardiovascular disease, hypertension, and certain cancers are prevalent among individuals with metabolic disorders, further complicating management and increasing mortality risk. For instance, individuals with T2DM are two to four times more vulnerable to develop cardiovascular diseases or renal failure compared to those without T2DM. Current trends in diabetology therefore emphasize a multitarget approach to managing diabetes by targeting metabolic syndrome alongside cardiovascular and renal complications. In particular, sodium-glucose cotransporter-2 (SGLT2) inhibitors and glucagon-like peptide-1 receptor agonists (GLP-1) receptor agonists show very promising results in the reduction of metabolic syndrome and its complications. These agents not only help manage blood glucose levels but also reduce the risk of cardiovascular events and delay the progression of renal disease. Additionally, lifestyle interventions, including dietary modifications, physical activity, and weight management, are critical components of comprehensive care, aiming to reduce the risk factors associated with metabolic syndrome (Knudsen and Lau, 2019; Chao and Henry, 2010; Díez-Manglano et al., 2014).

Although research indicates that individuals with diabetes are up to twice as likely to develop CPDs (Watz et al., 2009), the development of more effective therapies targeting pulmonary complications in patients with T2DM is often overshadowed by the emphasis on cardiorenal complications. Nevertheless, the respiratory issues have a significant impact on the patient’s health and prognosis. Increased systemic inflammation during pulmonary failure increases the risk of cardiovascular diseases by damaging blood vessels and contributing to plaque formation. Reduced oxygen levels in the blood (hypoxia) and elevated levels of carbon dioxide (hypercapnia) may lead to heart failure due to increased pressure in the pulmonary arteries. Finally, pulmonary diseases can affect the autonomic nervous system, which regulates heart rate and blood pressure. Dysregulation can lead to increased cardiovascular risks, including arrhythmias and hypertension (Forfia et al., 2013). Focusing on pulmonary health is therefore crucial for managing metabolic syndrome and its associated complications, highlighting the importance of comprehensive and integrated care approaches.

### 4.1 Impact of metabolic syndrome on the development of chronic respiratory diseases

T2DM can lead to mechanical changes in the respiratory system by imposing both metabolic (hyperglycemia, lipotoxicity) and vascular (microangiopathy) stress on pulmonary tissue. Pulmonary microangiopathy, a hallmark of diabetic vascular injury, reduces alveolar–capillary recruitment and impairs gas diffusion capacity, leading to decreased diffusing capacity for carbon monoxide, independently of confounding factors such as smoking or obesity (Hsia and Raskin, 2005). At the same time, chronic hyperglycemia promotes the formation of advanced glycation end-products (AGEs), which cross-link collagen and elastin fibers within the ECM. This process increases parenchymal stiffness, diminishes elastic recoil, and contributes to restrictive mechanical changes in the lung (Al-Robaiy et al., 2013). Clinical and epidemiological studies consistently demonstrate that individuals with T2DM exhibit reduced forced vital capacity (FVC), forced expiratory volume in one second (FEV_1_), and diffusion capacity, with the extent of decline correlating with glycemic burden and disease duration (Kinney et al., 2014).

Reduced lung volume, impaired diaphragm function and increased airway resistance can predispose individuals with metabolic syndrome to conditions such as obstructive sleep apnoea and obesity hypoventilation syndrome (Steier et al., 2008). T2DM is also associated with microvascular complications that can affect the pulmonary vasculature and lead to the development of hypoxia, pulmonary hypertension and an increased risk of pulmonary embolism or fibrosis (Jagadapillai et al., 2016; Roberts et al., 2018).

In addition, systemic inflammation, insulin resistance, and autonomic neuropathy further impair respiratory control and mechanical function, reinforcing the concept of the so-called “diabetic lung”. Chronic inflammation promotes oxidative stress and activates profibrotic signaling pathways, leading to ECM accumulation and progressive pulmonary dysfunction (Pradhan, 2001).

Furthermore, lipotoxicity resulting from insulin resistance and excessive circulating free fatty acids induces structural remodeling of the alveolar parenchyma and alters surfactant metabolism, contributing to both mechanical dysfunction and impaired gas exchange efficiency (Foster et al., 2010). Collectively, these mechanisms establish a multifactorial link between metabolic dysregulation in T2DM and progressive deterioration of pulmonary structure and function. However, despite the well-recognized contribution of metabolic disorders to respiratory morbidity, the effects of obesity on pulmonary function are not entirely uniform and appear to vary across different clinical settings and disease phenotypes. While excess adiposity may contribute to restrictive mechanical changes through reduced lung volumes and impaired diaphragmatic movement, several studies suggest that obesity is not uniformly associated with worse spirometric parameters across all patient populations (Alqarni et al., 2024). Moreover, in certain cohorts, particularly in patients with COPD, obesity has been associated with lower degrees of static hyperinflation and reduced emphysema severity (Dupuis et al., 2024), suggesting that adiposity may differentially affect distinct pulmonary phenotypes. These observations highlight the heterogeneity of obesity-related pulmonary manifestations and indicate that the impact of obesity on respiratory function likely depends on disease subtype, body fat distribution, and the balance between mechanical and inflammatory effects.

Finally, T2DM is linked to impaired function of immune cells such as neutrophils, macrophages and T-lymphocytes, leading to increased susceptibility to respiratory infections, including bacterial pneumonia, viral respiratory infections such as influenza and COVID-19, and fungal infections such as invasive pulmonary aspergillosis (Nojima et al., 2020). Beyond the direct effects of hyperglycemia on immune-cell function, metabolic dysfunction also influences immune regulation through mechanisms linked to nutritional immunity and the metabolic–inflammatory axis. Although nutritional immunity has traditionally been viewed as an evolutionarily conserved host-defense mechanism that limits nutrient availability during infection, similar adaptive responses may also occur in chronic metabolic disorders. Elevated glucose and free fatty acids may represent an evolutionarily conserved adaptive response that becomes maladaptive under persistent nutrient excess (Roth-Walter et al., 2024a). Chronic hyperglycemia and lipotoxicity promote activation of inflammatory pathways, including NF-κB signaling, oxidative stress responses, and innate immune activation, resulting in sustained low-grade inflammation (Jiao et al., 2009). Importantly, chronic inflammatory states alter the systemic distribution and bioavailability of micronutrients such as iron, copper, zinc, and selenium, which are essential regulators of mitochondrial function, redox balance, and immune responses (Balamurugan et al., 2024; Jomova et al., 2025). Disturbances in micronutrient homeostasis may further aggravate mitochondrial dysfunction and oxidative stress and amplify SASP signaling, which promote not only systemic inflammaging but also the accumulation and propagation of cellular senescence within the pulmonary system (Wiley et al., 2016; Ryan et al., 2023). Collectively, these mechanisms provide an additional framework linking metabolic dysfunction with immune dysregulation, chronic inflammation, and senescence-associated pulmonary injury.

The metabolic imbalance in patients with obesity and T2DM leads to the accumulation of senescent cells in the body. T2DM promotes cellular senescence in multiple tissues through the combined effects of metabolic and molecular stress. Chronic hyperglycemia and glucose fluctuations generate ROS and DNA damage, thereby activating p21^cip1/waf1^ and p16^INK4a^-dependent senescence pathways, as demonstrated in vascular endothelial cells (Maeda et al., 2015). The accumulation of AGEs further amplifies oxidative and inflammatory stress by engaging the receptor for AGEs (RAGE), reinforcing senescence-associated signaling (Cheng et al., 2023). In parallel, insulin resistance exacerbates metabolic imbalance by increasing circulating glucose and free fatty acids, which impair mitochondrial bioenergetics and promote endoplasmic reticulum (ER) stress. This lipotoxic environment, particularly elevated levels of saturated fatty acids, contributes to mitochondrial dysfunction and triggers senescence in diverse cell types—including endothelial, epithelial, and fibroblast populations in lungs (Dikalov et al., 2024; Zhang et al., 2025; Schmitt et al., 2024; Ceja-Galicia et al., 2025).The resulting SASP amplifies local inflammation, promotes aberrant tissue remodeling, and perpetuates lung dysfunction. These changes establish a direct link between metabolic dysregulation and structural alterations in the diabetic lung, thereby contributing to disease progression and poorer clinical outcomes. Accumulation of senescent cells in adipose tissue leads to disruption of adipose tissue homeostasis by increased lipid production and production of pro-inflammatory and pro-fibrotic molecules, thereby contributing to insulin resistance and systemic inflammation (Palmer et al., 2019). This inflammation acts as a contributing factor to both IPF and COPD in patients with obesity and T2DM.

Coronavirus disease 2019 (COVID-19), caused by the highly pathogenic SARS-CoV-2 virus, represents one of the most challenging health crises in the modern history. This disease is associated with high morbidity and mortality, primarily due to the development of severe acute respiratory syndrome. Numerous studies have shown that individuals with metabolic syndrome may be at higher risk for severe complications of COVID-19. T2DM and obesity have been significantly correlated not only with increased levels of SARS-CoV-2 viral particles detected in these individuals, but also with a worse course including hospitalization, the need for pulmonary ventilation, connection to extracorporeal circulation, and increased mortality. Other studies comparing mild and severe disease courses have identified the presence of metabolic syndrome as one of the key risk factors for severe outcomes.

SARS-CoV-2 infection triggers an increased release of pro-inflammatory cytokines and chemokines, which play a pivotal role in the development of pulmonary fibrosis. This results in an increased viral load for the patient, loss of lung function, lung damage and subsequently higher mortality. Senescent cells, which accumulate more abundantly in individuals with metabolic syndrome, play a significant role in the severity of the course of COVID-19 in patients with obesity and T2DM. In addition to their influence on impaired immune cell function, chronic inflammation, and reduced tissue regeneration, the presence of senescent cells may have a direct impact on the development of COVID-19. Some studies have shown that senescent cells accumulating in visceral adipose tissue increase the expression of angiotensin converting enzyme 2 (ACE2), which serves as a receptor for SARS-CoV-2 virus entry into the cell, where it then multiplies and accumulates. Consequently, expanded adipose tissue may act as a reservoir for SARS-CoV-2, thereby contributing to disease severity and poorer clinical outcomes in patients with metabolic syndrome (The Novel Coronavirus Pneumonia Emergency Response Epidemiology Team, 2020; Denson et al., 2021).

### 4.2 Impact of chronic respiratory diseases on the development of metabolic diseases

While there is limited evidence suggesting that IPF directly contributes to the development of metabolic syndrome, increasing evidence indicates that metabolic syndrome and its associated abnormalities, including obesity, insulin resistance, chronic inflammation, and oxidative stress, may contribute to the development and progression of IPF through mechanisms involving epithelial injury and dysregulated tissue repair (Kim et al., 2025). Persistent metabolic dysregulation can further promote mitochondrial dysfunction and activation of profibrotic signaling pathways, thereby facilitating pulmonary fibrotic remodeling (Bueno et al., 2015). These findings suggest that metabolic alterations are more likely to act as contributors to IPF pathogenesis rather than consequences of the disease itself.

In contrast, a high risk of developing metabolic syndrome has been detected in patients diagnosed with COPD. Studies show that up to 47% of patients with advanced COPD suffer from metabolic syndrome, which can have a fatal impact on an already weakened body (Watz et al., 2009). The most common metabolic diseases that accompany COPD include T2DM, obesity, dyslipidaemia, hypertension, hyperglycemia and increased risk of cardiovascular disease (Archer and Baker, 2009). The development of metabolic syndrome in patients with advanced COPD is linked to chronic pulmonary inflammation, which can trigger systemic inflammatory responses that contribute to insulin resistance and impaired glucose metabolism. This can also be affected by corticosteroids, which are often prescribed to patients. Shortness of breath, decreased appetite, eating difficulties or increased energy expenditure due to labored breathing in COPD patients may lead to weight loss or malnutrition, which affects glucose metabolism and increases the risk of T2DM (Zafar et al., 2010; Mannino et al., 2008).

In addition to the specific characteristics of COPD and metabolic syndrome patients, there is a close interrelationship between the two conditions that can lead to a deterioration in the patient’s overall condition. Besides genetic predisposition, common risk factors for COPD and metabolic syndrome include physical inactivity, unhealthy diet and the accumulation of cellular senescence. These factors interact and contribute to the development of both diseases. Chronic inflammation can mutually reinforce one another, further worsening the prognosis and reducing the quality of life. Moreover, COPD limits physical activity and contributes to the obesity and development of insulin resistance. Therefore, patients with COPD and metabolic syndrome are at significantly higher risk of death than those with COPD alone (Díez-Manglano et al., 2014; Mannino et al., 2008). For these reasons, comprehensive therapy that includes management of pulmonary symptoms in COPD, control of metabolic factors, and prevention and treatment of associated complications is necessary to improve the quality of life and survival of these patients.

## 5 Senolytic agents

Given the central role of cellular senescence in aging and age-related diseases, like metabolic syndrome and pulmonary diseases, there has been considerable interest in developing therapeutic strategies to target senescent cells. One such approach is the use of senolytic or senomorphic drugs. Senolytic drugs are therapeutic agents that selectively eliminate senescent cells from the organism, thereby reducing their accumulation and associated detrimental effects on tissue function. Unlike senolytics, senomorphic agents do not directly eliminate senescent cells. Their primary mechanism involves modulation of the senescent phenotype through suppression of SASP production and restoration of cellular homeostasis. By reducing oxidative stress, improving mitochondrial function, and modulating nutrient-sensing pathways, some senomorphic agents may also delay the progression of senescence under specific conditions (Zhu et al., 2015).

Most of the currently tested senolytic agents target specific pathways that protect senescent cells from programmed cell death. One of the main mechanisms involves targeting specific enzymes involved in anti-apoptotic processes, including p53, p21^cip1/waf1^, BCL-2 proteins, the PI3K/Akt pathway, SIRT1, and FOXO4. Senomorphic agents, on the other hand, primarily suppress the production of SASP by targeting signaling pathways such as NF-κB, mTOR (mechanistic Target Of Rapamycin), IL-1α, and p38/MAPK (Zhang et al., 2023). The reduction of senescence through senolytic and senomorphic agents can thus alleviate degenerative processes in the body and improve the health and quality of life of patients with CPDs and metabolic syndrome (Wissler Gerdes et al., 2020; Kirkland and Tchkonia, 2020).

Currently, many senolytic agents are being tested in preclinical studies, some of which have advanced to various phases of clinical testing. One of the most promising senolytics is the combination of dasatinib and quercetin (D + Q). Dasatinib is a tyrosine kinase inhibitor approved by the Food and Drug Administration (FDA), which effectively suppresses cell proliferation and migration, activates apoptosis, and is used in cancer treatment. Quercetin is a natural flavonoid with diverse biological activities. This combination was identified as the first senolytic therapy that promotes apoptosis of senescent cells by targeting the PI3K/AKT pathway (Zhu et al., 2015). These substances alone have not shown significant effects, but their combination demonstrates high efficacy in eliminating senescent cells. The D + Q combination is effective in various age-related diseases, including IPF, where a positive effect of D + Q on reducing pulmonary fibrosis and improving respiratory function has been demonstrated (Wissler Gerdes et al., 2020). In relation to this disease, phase 1 of clinical trial is currently underway (study registration number: NCT02874989) (Justice et al., 2019). Other studies have demonstrated a beneficial effect of dasatinib and quercetin (D + Q) in reducing kidney damage, where elimination of senescent cells leads to improved renal structure and function (Zhu et al., 2024). The use of this treatment in patients with chronic kidney damage is currently being tested in phase 2 of clinical trial (study registration number: NCT02848131) (Hickson et al., 2019). Liver steatosis and osteoporosis belong to the other age-related diseases being studied in preclinical trials with D + Q. Although D + Q is currently among the most clinically advanced senolytic strategies, further studies are needed to optimize treatment schedules and better characterize potential adverse effects associated with dasatinib, including pleural effusions, fluid retention, and myelosuppression, which have been reported in oncology patients receiving long-term dasatinib treatment (Cortes et al., 2016).

Another natural flavonoid related to quercetin is fisetin, which has antioxidant properties and is therefore being studied for its anti-senescent potential. Preclinical research has shown that fisetin alleviates systemic inflammation, SASP, and reduces senescent markers in aged mice, which was associated with an extension of lifespan in these experimental models (Yousefzadeh et al., 2018). Although fisetin demonstrated senolytic activity and lifespan-extending effects in preclinical models, clinical evidence remains limited. Several early-phase human studies are currently evaluating its safety, feasibility, and potential effects on aging-related outcomes (AFFIRM-LITE study; NCT03675724). However, its senolytic efficacy, optimal dosing, and long-term clinical benefits in humans remain to be established. Other studies suggest that fisetin alleviates podocyte damage induced by diabetic nephropathy (Dong et al., 2022), and enhances the antioxidant defense of the hearts in diabetic animals (Althunibat et al., 2019). In this context, phase 2 of clinical testing is currently underway to assess the effect of senescent cell elimination on improving cardiovascular function (study registration number: NCT06133634) (Matthew Rossman, 2023).

BCL-2 family inhibitors, particularly navitoclax (ABT-263), represent an emerging class of senolytic agents with potential relevance for chronic pulmonary diseases characterized by the accumulation of apoptosis-resistant senescent cells. Senescent epithelial cells, fibroblasts, and endothelial cells in IPF and COPD exhibit upregulated anti-apoptotic BCL-2 family proteins, which enable their persistence and sustained production of SASP factors that drive chronic inflammation and fibrotic remodeling of the lung. Pharmacological inhibition of BCL-2/BCL-xL pathways selectively induces apoptosis of these senescent cells, thereby reducing senescent cell burden, attenuating extracellular matrix deposition, and improving tissue regenerative capacity in preclinical models of pulmonary fibrosis and emphysema (Pan et al., 2017). Although clinical evidence in respiratory diseases remains limited, the initiation of early clinical evaluation of BCL-2–targeting strategies, including a phase I trial of the BCL-2 inhibitor venetoclax in idiopathic pulmonary fibrosis (NCT05976217), supports the translational potential of targeting anti-apoptotic survival pathways. However, the clinical translation of navitoclax still remains limited by dose-dependent thrombocytopenia resulting from BCL-xL inhibition in platelets. The development of next-generation BCL-2 family inhibitors and more selective senolytic approaches may help overcome this limitation while preserving therapeutic efficacy (Mason et al., 2007; Wilson et al., 2010).

Rapamycin, also known as a sirolimus, is a macrolide compound with immunosuppressive and anti-proliferative properties that has been extensively studied as a senomorphic agent. Its anti-senescent effects are primarily mediated through inhibition of the mTOR pathway, a central regulator of cellular growth, metabolism, and senescence (Johnson et al., 2013). By suppressing mTOR signaling, rapamycin reduces the production of SASP factors, attenuates chronic inflammation, and slows the progression of senescence-associated cellular dysfunction (Herranz et al., 2015; Laberge et al., 2015). In addition, rapamycin has been shown to extend lifespan and improve healthspan in preclinical models, further supporting its potential as a senescence-modulating therapeutic strategy (Harrison et al., 2009).

Although senolytic and senomorphic approaches have shown encouraging results in preclinical studies (summarized in Table 1), their translation into clinical practice remains challenging. Human studies conducted to date are limited in size and duration, and reported therapeutic benefits have often been modest or variable across patient populations, with some outcomes showing effects comparable to placebo (Roth-Walter et al., 2024b). In addition, concerns regarding potential adverse effects, including off-target toxicity and interference with physiological functions of senescent cells, warrant cautious interpretation of current findings. Therefore, larger, well-controlled clinical trials are needed to establish the efficacy and long-term safety of these therapeutic strategies (Nambiar et al., 2023). An additional challenge is the context-dependent role of cellular senescence in tissue remodeling. While persistent accumulation of senescent cells contributes to chronic inflammation and fibrosis, transient senescence may also participate in physiological wound-healing responses (Demaria et al., 2014). Consequently, the timing of senolytic intervention may be critical, and further studies are required to determine the optimal therapeutic window for senescent-cell clearance in chronic pulmonary diseases.

**TABLE 1:** Overview of senolytic and senomorphic agents relevant to chronic pulmonary diseases, including their molecular targets, mechanisms of action, and current evidence level.

Agent	Type	Main target/Mechanism	Proposed effects relevant to pulmonary diseases	Current evidence	Clinical development
Dasatinib + quercetin (D + Q)	Senolytic	Tyrosine kinase inhibition, PI3K/AKT-related anti-apoptotic pathways	Elimination of senescent cells, reduced fibrosis and inflammation, improved tissue function	Preclinical + early clinical	Phase I IPF (NCT02874989); phase II diabetic kidney disease (NCT02848131)
Fisetin	Senolytic	Anti-inflammatory activity, modulation of oxidative stress and survival pathways	Reduced SASP, decreased inflammation, improved vascular and metabolic function	Mainly preclinical + early clinical	Phase II cardiovascular/aging-related studies (NCT06133634)
Navitoclax (ABT-263)	Senolytic	BCL-2/BCL-xL inhibition	Selective apoptosis of senescent cells, reduced fibrosis and ECM deposition	Mainly preclinical	Limited early clinical studies
Venetoclax	Senolytic	BCL-2 inhibition	Targeting apoptosis-resistant senescent cells	Early translational evidence	Phase I IPF (NCT05976217)
Rapamycin (sirolimus)	Senomorphic	mTOR inhibition	Suppression of senescence-associated pathways and SASP	Preclinical + clinical	Multiple clinical studies in aging-related conditions

### 5.1 Anti-diabetic therapies with senolytic effect

As mentioned above, CPDs are increasingly recognized in patients with metabolic syndrome highlighting the need for integrated management strategies. Since the effect of senescent cell accumulation on the development of both CPDs and metabolic syndrome has been demonstrated, several anti-diabetic therapies with a potential effect on the reduction of senescent cells have been detected (Table 2).

**TABLE 2:** Overview of anti-diabetic therapies with senolytic effect, including their molecular targets, mechanisms of action, and current evidence level.

Agent	Type	Main target/Mechanism	Proposed effects relevant to pulmonary/Metabolic diseases	Current evidence	Clinical development
Metformin	Senomorphic	AMPK activation, mitochondrial complex I inhibition, mTOR modulation	Reduced inflammation and oxidative stress, suppression of senescence-associated pathways	Preclinical + clinical	Multiple clinical studies including TAME and MILES
GLP-1 receptor agonists (e.g., liraglutide, semaglutide)	Senomorphic-like	GLP-1 receptor activation; AMPK activation, improved mitochondrial function and anti-inflammatory signaling	Reduced oxidative stress, attenuation of SASP, reduced cellular senescence burden, improved cardiometabolic and pulmonary outcomes	Preclinical + emerging clinical evidence	Multiple approved clinical applications (T2DM, obesity, cardiovascular risk reduction)
Dapagliflozin	Senomorphic-like	SGLT2 inhibition	Reduced oxidative stress and glucose-induced senescence	Preclinical + clinical	Approved for T2DM/CVD/HF
Empagliflozin	Senomorphic-like	SGLT2 inhibition, AMPK-related pathways	Reduced SASP, improved mitochondrial function	Preclinical + clinical	Approved for T2DM/CVD/HF
Canagliflozin	Senomorphic-like	SGLT2 inhibition	Reduced adipose senescence burden and metabolic dysfunction	Mainly preclinical + clinical	Approved for T2DM/CVD/HF
Tirzepatide	Senomorphic-like	Dual GIP/GLP-1 receptor agonist; improves insulin sensitivity, reduces inflammation and metabolic stress	Reduced oxidative stress and chronic inflammation, attenuation of SASP-related pathways, improved mitochondrial function and cardiometabolic status, potential indirect reduction of senescent-cell burden	Preclinical + emerging clinical evidence	Approved for T2DM and obesity; multiple cardiovascular and metabolic outcome trials

#### 5.1.1 Metformin

Metformin is an approved and currently one of the most widely used drugs for the treatment of T2DM. In addition to its hypoglycemic effect mediated by inhibition of glucose absorption, increased peripheral insulin sensitivity and reduced glucose synthesis, metformin has a beneficial effect on metabolic and cellular processes closely associated with the development of age-related conditions such as inflammation and cellular senescence (Martin-Montalvo et al., 2013).

One of the described mechanisms of action of metformin in eliminating senescent cells involves inhibition of mitochondrial complex I (CI), a key enzyme complex involved in cellular respiration and ATP production. This reduction in ATP levels induces activation of AMP-activated protein kinase (AMPK), which subsequently activates cell death in senescent cells (Owen et al., 2000). Launched in 2018, the TAME (Targeting Aging with Metformin; study registration number: NCT02432287) clinical trial aims to show that metformin targets aging while preventing the development of age-related complications. Another aim of the study includes measuring physical and cognitive function and quality of life in individuals (Albert Einstein College of Medicine and N, 2021).

Activation of AMPK by metformin also inhibits TGFβ-induced NOX4 expression and concomitantly decrease ROS production in lung fibroblasts, resulting in the amelioration of senescence and lung fibrosis development (Sato et al., 2016). Moreover, mice with diet-induced obesity treated with metformin showed decreased lung inflammation, emphysematous change, and airway remodeling. Additionally, oxidative stress, cell death, telomere attrition, and cellular senescence were suppressed (Polverino et al., 2021). Finally, it was described that metformin attenuates cigarette smoke extract-induced cellular senescence by suppressing mTOR signaling (Saito et al., 2019).

Although metformin exhibits some senescence-reducing effects, it was not originally developed as a senolytic agent, and its activity is likely less specific than that of targeted senolytics. The optimal dose and treatment duration for achieving a senolytic response remain undefined. Careful dose optimization is crucial, as higher doses may cause gastrointestinal side effects or, in rare cases, lactic acidosis, particularly in patients with renal impairment (Raičević and Janković, 2023).

#### 5.1.2 SGLT2 inhibitors

SGLT2 inhibitors are a novel class of anti-diabetic drugs targeting glucose reabsorption in the kidneys. By inhibiting SGLT2-mediated renal glucose reabsorption, these agents induce glycosuria and lower blood glucose independently of insulin. This mechanism reduces the risk of hyperglycemia and contributes to weight loss and blood pressure improvement. Developed from insights into the effects of phlorizin and genetic conditions such as familial renal glycosuria, SGLT2 inhibitors represent a new approach to leveraging renal glucose regulation in managing T2DM (Chao and Henry, 2010; Bhanot Sea, 2009; Bakris et al., 2009). However, it has recently been shown that SGLT2 inhibitors have also a beneficial effect on the reduction of senescent cells in the organism.

##### 5.1.2.1 Dapagliflozin

Dapagliflozin is a highly selective SGLT2 inhibitor particularly effective in alleviating glucotoxicity, supporting β-cell function, and improving insulin sensitivity, making it a suitable option for managing T2DM with insulin resistance or β-cell dysfunction (Meng et al., 2008; Han et al., 2008).

By reducing oxidative stress and inflammation while improving mitochondrial function, dapagliflozin may attenuate processes that promote cellular senescence. It also modulates autophagy pathways, which are crucial for cellular cleanup and rejuvenation, potentially aiding in the clearance of damaged or senescent cells (Zhou et al., 2023).

It is well described that dapagliflozin reduces atherosclerosis, lowers blood pressure and impaired endothelium-dependent vasorelaxation in patients with T2DM. Beyond its role in improving metabolic parameters, dapagliflozin has been shown to restore SIRT1 signaling in endothelial cells, thereby preventing the accumulation of senescent cells in the vascular endothelium of patients with T2DM and reducing the risk of diabetic vascular complications (Tai et al., 2023). Although direct evidence in CPDs is currently lacking, these findings suggest that similar mechanisms may also be relevant in pulmonary diseases characterized by endothelial dysfunction and cellular senescence.

Dapagliflozin offers renal protection in subjects with T2DM by reducing hyperfiltration, lowering albuminuria, and mitigating oxidative stress and inflammation in renal tissue. The decreased influx of glucose into renal tubular epithelial cells prevents high-glucose-induced cellular senescence. Since cellular senescence contributes to the pathogenesis of diabetic nephropathy, delineating the related molecular mechanisms and the effects of the widely used gliflozins on them is of particular interest and may lead to novel therapeutic approaches (Eleftheriadis et al., 2022).

##### 5.1.2.2 Empagliflozin

Compared to dapagliflozin, empagliflozin is a highly selective SGLT2 inhibitor that promotes urinary glucose excretion while sparing SGLT1 activity, reducing the risk of hypoglycemia and gastrointestinal side effects. It lowers glycated hemoglobine, supports β-cell function, and contributes to weight loss, enhancing glycemic control and metabolic outcomes (Anadu Ndefo et al.). Empagliflozin also exerts glucose-independent benefits, such as reducing diabetic neuropathy and oxidative stress through AMPK pathway modulation. These effects make empagliflozin a promising therapeutic candidate for addressing complications beyond glycemic control, particularly those related to inflammation and vascular dysfunction (Scott, 2014; Eid et al., 2020).

By reducing hyperglycemia and associated oxidative stress, it mitigates conditions that promote senescent cell accumulation. Empagliflozin enhances mitochondrial function and supports autophagy, aiding the clearance of damaged and senescent cells (Mattii et al., 2024; Zhou et al., 2018). Additionally, it alleviates the pro-inflammatory SASP, reducing systemic inflammation and tissue dysfunction. Experimental studies have shown that empagliflozin reduces the senescence of cardiac stromal cells and improves cardiac function in a murine model of diabetes (Madonna et al., 2020). Empagliflozin has also been reported to reduce hepatic senescent-cell burden and extend lifespan in naturally aged mice (Long et al., 2024). Although no direct effect on lung function has been described, the EMBRACE-HF trial showed decrease in pulmonary artery pressure in patients with heart failure treated with empagliflozin (Nassif et al., 2021). By lowering pulmonary artery pressure, empagliflozin may indirectly mitigate processes associated with pulmonary cellular senescence.

##### 5.1.2.3 Canagliflozin

Besides inhibition of renal glucose reabsorption, canagliflozin lowers blood pressure *via* osmotic diuresis, making it particularly useful in patients with T2DM and hypertension. These benefits extend to cardiovascular protection, as canagliflozin reduces the risk of major cardiovascular adverse events (Singh et al., 2014; No et al., 2010). When combined with metformin, canagliflozin improves glycemic control, weight management and cardiovascular outcomes, supporting its use in patients with high cardiovascular risk.

In ApoE-deficient mice, canagliflozin reduced atherosclerotic burden, an effect that correlated with a lower senescent-cell burden (Katsuum et al., 2023). Moreover, in a mouse model of dietary obesity, treatment with the canagliflozin reduced the senescence load in visceral adipose tissue and improved adipose tissue inflammation and metabolic dysfunction as well as prolonged lifespan of the animals. It has been reported that canagliflozin promotes the clearance of senescent cells by activating immune responses through the downregulation of PD-L1 expression (Katsuumi et al., 2024).

Collectively, preclinical studies indicate that canagliflozin can substantially reduce senescent-cell burden and improve tissue homeostasis, supporting its potential relevance for age-related diseases, including CPDs.

#### 5.1.3 GLP-1 agonists

GLP-1 receptor agonists, such as liraglutide and semaglutide, play a crucial role in managing T2DM and related metabolic conditions. These medications mimic the GLP-1 hormone to enhance glucose-dependent insulin secretion, inhibit glucagon release, delay gastric emptying, and increase satiety. Their effects extend beyond glycemic control to include significant weight reduction and cardiovascular improvement. They reduce inflammation markers, such as CRP and IL-6, which contribute to improved vascular health and stabilisation of atherosclerotic plaques (Knudsen and Lau, 2019).

Research across organisms suggests that activating GLP-1 receptors significantly impacts cellular processes linked to aging. Improved mitochondrial function, enhanced cellular stress resistance, and reduced inflammation suggest that GLP-1 receptor activation may influence fundamental mechanisms of aging beyond its metabolic actions (Peng et al., 2022). GLP-1 receptor agonists suppress oxidative stress and inflammatory signaling, including the NF-κB pathway, a key regulator of cellular senescence. By modulating cytokine profiles and reducing markers of cellular damage, GLP-1 receptor agonists contribute to decreased senescence-associated phenotypes in pulmonary and systemic conditions (Janić et al., 2024).

#### 5.1.4 Tirzepatide

Tirzepatide is an innovative dual agonist that simultaneously activates GIP and GLP-1 receptors. Its mechanism enhances insulin secretion, suppresses glucagon production, slows gastric emptying, and reduces appetite, leading to improved glycemic control and significant weight loss. These effects address the challenges of hyperglycemia and obesity, common in individuals with T2DM (Forzano et al., 2022). Moreover, tirzepatide also suppresses inflammatory markers and improves cardiovascular parameters, including reductions in systolic blood pressure and LDL cholesterol levels (Kanbay et al., 2023).

Recent studies suggest that tirzepatide may influence cellular senescence. By improving metabolic homeostasis, reducing systemic inflammation, and enhancing mitochondrial function, tirzepatide can mitigate the conditions that promote senescent cell accumulation. It may indirectly facilitate the elimination of senescent cells through its impact on autophagy, oxidative stress, and insulin sensitivity. These effects could contribute to delaying age-related pathologies.

Although several antidiabetic agents exhibit senescence-modulating properties, the underlying mechanisms remain incompletely understood. Their effects may vary among different senescent-cell populations, highlighting the need to identify shared molecular vulnerabilities that could serve as broadly applicable therapeutic targets in metabolic and age-related diseases.

### 5.2 The impact of current CPDs therapies on cellular senescence

Some medications currently used in the treatment of CPD may have indirect effects on senescence. Drugs such as corticosteroids, antifibrotics, a phosphodiesterase 4 inhibitor (PDE4), antioxidants and certain antibiotics may modulate senescence indirectly by influencing inflammation, oxidative stress, or pathways associated with the SASP. Although these drugs are not designed as senolytics, their impact on senescence opens up opportunities for further research in CPDs treatment. The previously mentioned antifibrotics, such as pirfenidone and nintedanib, specifically target fibrotic processes and inflammation in the lungs. These drugs interfere with pathways like TGF-β, VEGF, and PDGF, which are involved in maintaining senescence. As a result, they may reduce the activity of senescent cells and their contribution to the fibrotic environment (Roth et al., 2015).

In patients with more severe forms of COPD, inhaled corticosteroids are commonly used. Corticosteroids can modulate inflammation, which is a key factor in the induction and maintenance of senescence. They suppress pro-inflammatory cytokines, such as IL-6 and TNF-α, which are components of the SASP. However, long-term use of corticosteroids can induce senescence in fibroblasts and epithelial cells in the lungs, potentially contributing to tissue remodeling and worsening some pulmonary conditions. Moreover, corticosteroids also modulate the function of immune cells. Prolonged corticosteroid exposure can alter the phenotype and activity of macrophages, T-lymphocytes, and neutrophils, potentially impairing clearance of senescent cells and disrupting the balance between pro-inflammatory and reparative immune responses. This immunomodulatory effect may further contribute to tissue remodeling and chronic inflammation in the lungs. Therefore, considering the role of immune cell–senescence interactions could provide a more comprehensive understanding of how long-term corticosteroid therapy influences pulmonary pathology (Fernandes et al., 2022).

When basic treatment in COPD patients is insufficient, roflumilast, a PDE4 inhibitor, is often prescribed. Roflumilast reduces the production of pro-inflammatory mediators and suppresses systemic inflammation, which can reduce the accumulation of senescent cells (Rabe, 2011).

It is important to note that currently available therapies for CPDs were not primarily developed to target cellular senescence, and their indirect senescence-modulating effects remain insufficiently characterized. Among currently investigated senolytic approaches, the combination of dasatinib and quercetin (D + Q) appears particularly promising due to its ability to selectively eliminate senescent cells through complementary targeting of pro-survival pathways. In contrast to agents acting predominantly through indirect anti-inflammatory or metabolic effects, D + Q demonstrated consistent senolytic activity across multiple experimental models of fibrosis, metabolic dysfunction, and age-related tissue degeneration, including pulmonary fibrosis (Schafer et al., 2017; Lehmann et al., 2017). Moreover, D + Q is currently among the most clinically advanced senolytic strategies, with early clinical studies indicating acceptable feasibility and preliminary evidence of improved physical function and reduced senescence-associated burden in humans (Justice et al., 2019; Nambiar et al., 2023). Nevertheless, although several early clinical studies for D + Q and other agents already evaluate feasibility, safety, and selected efficacy endpoints of senolytic or senomorphic interventions, these trials are generally limited in size, duration, and disease specificity. Therefore, further studies are still needed to define long-term safety, repeated-dosing effects, validated biomarkers of senescent-cell burden, and clinically meaningful pulmonary outcomes.

From a clinical perspective, senolytic therapies are most likely to benefit patient populations characterized by accelerated accumulation of senescent cells, chronic low-grade inflammation, and progressive tissue remodeling, but who still retain sufficient regenerative capacity to respond to treatment. In pulmonary medicine, the most promising target groups may therefore include patients with early-to-moderate IPF, progressive COPD with emphysematous remodeling, and individuals with obesity- or T2DM-associated pulmonary dysfunction accompanied by systemic inflammaging and metabolic stress. In these patients, senescence contributes to chronic inflammation, impaired tissue repair, fibrosis, endothelial dysfunction, and immune dysregulation, suggesting that selective elimination of senescent cells could slow disease progression before irreversible structural damage predominates [summarized in (Barnes et al., 2019).

In contrast, patients with end-stage pulmonary fibrosis, severe cachexia, advanced frailty, extensive organ failure, or profound immunosenescence may derive more limited benefit, as irreversible tissue destruction and reduced regenerative potential could diminish the efficacy of senescence-targeted interventions (Nov et al., 2021). Early-phase clinical studies should therefore preferentially focus on clinically stable patients with evidence of elevated senescence burden or SASP activation, but without severe immunosuppression, active infection, impaired wound healing, or major hematological toxicity risk.

The success of such approaches will strongly depend on the identification and validation of reliable biomarkers enabling patient stratification, monitoring of therapeutic response, and assessment of senescent-cell burden. Markers of senescence detectable in plasma primarily include circulating components of the senescence-associated secretory phenotype (SASP), inflammatory cytokines, chemokines, growth factors, proteases, and markers of tissue remodeling. Frequently studied plasma biomarkers comprise IL-6, IL-1β, TNF-α, MCP-1 (CCL2), TGF-β, GDF15, PAI-1, MMPs (particularly MMP-1, MMP-3, and MMP-9), VEGF, and CRP (Shin et al., 2022). Additional circulating markers associated with senescence include extracellular vesicles, cell-free mitochondrial DNA, oxidative stress markers, and molecules reflecting mitochondrial dysfunction or impaired proteostasis (Picca et al., 2019). More recently, multi-marker plasma panels integrating SASP-associated proteins with markers of biological aging and systemic inflammation have emerged as promising tools for minimally invasive assessment of systemic senescent-cell burden and monitoring of senescence-targeted therapies (Tanaka et al., 2018).

Collectively, these findings highlight the growing therapeutic potential of senescence-targeted interventions in chronic pulmonary and metabolic diseases. Although current evidence remains largely preclinical or based on early-phase clinical studies, ongoing advances in senolytic therapies, biomarker development, and patient stratification may enable more precise and mechanism-based approaches for the treatment of CPDs in the future.

## 6 Conclusion

The prevalence of CPDs not only in patients with metabolic diseases is steadily increasing worldwide, mainly due to the aging population and the lack of effective methods to reduce risk factors associated with the development/progression of these diseases. Despite extensive research in recent years that has yielded many new insights into the pathogenesis of these diseases, there is currently still a lack of effective therapies that could potentially be used to treat and/or prevent CPDs, which, in addition to the negative impact on the patient, also increases the health and economic burden on society. The current treatment of patients with chronic lung injury primarily involves palliative care to alleviate discomfort and delay lung failure.

Although there is currently no direct treatment targeting the cause of these diseases, there is a new potential strategy that focuses on the selective elimination of senescent cells as one of the main causes of lung damage and development of CPDs. Senolytics therefore represent great potential in the treatment of age-related diseases and pose a challenge for research into future effective therapies, not only for CPDs but for a wider range of age-associated conditions.
